# Comparative study of growth performance, nutrient digestibility, and ruminal and fecal bacterial community between yaks and cattle-yaks raised by stall-feeding

**DOI:** 10.1186/s13568-021-01259-9

**Published:** 2021-06-30

**Authors:** Qindan Dai, Jian Ma, Guang Cao, Rui Hu, Yixiao Zhu, Guangyang Li, Huawei Zou, Zhisheng Wang, Quanhui Peng, Bai Xue, Lizhi Wang

**Affiliations:** 1 Low Carbon Breeding Cattle and Safety Production University Key Laboratory of Sichuan Province, Animal Nutrition Institute, Sichuan Agricultural University, Chengdu, 611130 China; 2 College of Animal Science, Xinjiang Agricultural University, Urumchi, 830052 China

**Keywords:** Yak, Cattle-yak, Nutrient digestibility, Rumen fermentation, Bacterial community

## Abstract

The experiment was conducted to compare the growth performance, rumen fermentation, nutrient digestibility, and ruminal and fecal bacterial community between yaks and cattle-yaks. Ten male yaks (36-month-old) were used as the yak (YAK) group and 10 male cattle-yaks with similar age were selected as the cattle-yak (CAY) group. All the animals were fed same ration and the experiment lasted for 60 days. The results showed that the average daily gain and dry matter intake of CAY group were higher (*P* < 0.05) than those of YAK group. The ruminal concentrations of total volatile fatty acids, acetate, and butyrate were higher (*P* < 0.05) in CAY group than those in YAK group. However, the neutral detergent fiber and acid detergent fiber digestibility exhibited an opposite between two groups. In the rumen, the relative abundances of *Prevotella 1* and *Prevotellaceae UCG-001* were higher (*P* < 0.05) and *Succiniclasticum* and *Butyrivibrio 2* were lower (*P* < 0.05) in YAK group compared to CAY group. In the feces, *the unclassified Lachnospiraceae*, *Lachnospiraceae NK4A136 group*, and *Lachnospiraceae AC2044 group* were significantly enriched (*P* < 0.05) in YAK group, whereas the *Ruminococcaceae UCG-010*, *Ruminococcaceae UCG-013*, and *Succiniclasticum* were significantly enriched (*P* < 0.05) in CAY group. Overall, under the same diet, the yaks have higher fiber utilization and cattle-yaks have higher energy utilization.

**Supplementary Information:**

The online version contains supplementary material available at 10.1186/s13568-021-01259-9.

## Introduction

The yak (*Bos grunniens*) is an indigenous ruminant that can adapt to the extremely harsh environment on the Qinghai-Tibetan Plateau. The Qinghai-Tibetan Plateau is characterized by high altitude, low temperature, strong ultraviolet light, and severe cold. Most of yaks are distributed in China, and the yak provides the major living resources, including fur, fuel (feces for living fuel), milk, and meat for the local Tibetan herdsmen (Ma et al. 2020b). The yak remains semi-domesticated status, pasturing on the meadow with a natural mating, and has a vital ecological niche in the Qinghai-Tibetan Plateau ecosystem (Zhang et al. 2016). However, the climatic environment of Qinghai-Tibetan Plateau is sharp frost (average temperature − 15 to − 5 ℃) in the long cold season (from October to May). Due to the withered and snow-covered grass, the forage is extremely limited in the cold season. Thus, the energy and protein intake are not enough for the maintenance requirement of yaks. Generally, in the cold season, the yaks can lose 25% of body weight (BW) (Long et al. 2004). The production performance of yaks cannot catch up with beef cattle, which leads to lower economic income.

After long-term natural selection, the ruminal microbiota of yaks have higher ability of fiber degradation (Ma et al. 2020c). In order to improve the production performance of yaks on the plateau, the cattle-yak, which has unique nutrient degradation capability, is bred to combine the tolerance of cold and high altitudes of yaks with the excellent productivity of other cattle (Liao et al. 2018). The cattle-yaks are well adapted to the harsh environment of plateau and display higher growth performance compared to yaks. At present, the yaks and cattle-yaks are widely pastured on the plateau in China, but the feed efficiency of yaks and cattle-yaks is lower. Most of yaks and cattle-yaks are slaughtered without fattening process, which reduce the production performance of animals.

Breed is an important factor that can affect the production performance, rumen fermentation, nutrient digestibility, and ruminal microbiota of ruminants (Carroll et al. 2011; Zhu et al. 2020). Ruminal microbiota directly or indirectly contributes to the animal production. A previous study has compared the ruminal microbiota of three beef cattle breeds by using metagenomics and transcriptomics, and found that the ruminal microbiota of three breeds was different in microbial populations or digestive enzyme activities (Li et al. 2019a). The ruminal microbiota, which plays an vital role in the rumen fermentation, is composed of bacterial, archaea, fungi, and protozoa (Morgavi et al. 2013). Xin et al. (2019) reported that the dominant bacteria of rumen were distinct in different cattle breeds, and the *Prevotella* and *Succiniclasticum* were prevalent in yellow cattle, whereas the *Christensenellaceae R7 group* and *Lachnospiraceae UCG 008 group* were abundant in yaks. Based on previous studies mentioned earlier, we found that breed is a natural factor that can affect the feed utilization and ruminal microbiota. However, under the same diet, the differences of production performance and gut microbiota of yak and cattle-yak are still unclear.

In the current study, we hypothesized that the cattle-yaks had the heterosis and might display higher growth performance which may be attributed to the difference of microbiota under the same diet. Therefore, we investigated the differences of growth performance, rumen fermentation, nutrient digestibility, serum biochemical indexes, ruminal and fecal microbiota between yaks and cattle-yaks under the same diet. These results will be important for understanding the role of microbiota in the nutrition and metabolism of yaks and cattle-yaks. Moreover, the results will provide experimental basis for the rational design and scientific raising of yaks and cattle-yaks.

## Materials and methods

### Experimental design, diet, and management

This experiment was conducted at Hongyuan Experimental Station of Grassland Research Institute, Sichuan Province, China (altitude approximately 3500 m; 32°48′N latitude and 102°33′E longitude). Ten male Maiwa yaks (36-month-old) were used as the yak (YAK) group. Another 10 male cattle-yaks (♀Maiwa yak × ♂Simmental cattle) with the similar age were selected as the cattle-yak (CAY) group. Animals in the two groups were housed in 20 pens with 1 animal in each pen. All the cattle were provided rations twice daily at 08:00 and 16:00 and had free access to water.

Before experiment, all cattle were marked with ear tags; meanwhile, parasites were expelled. A 10-d adaptive phase was followed by 60 d of experimental period. All cattle were fed with the same total mixed ration, which was formulated according to the Chinese Beef Cattle Raising Standard (NY/T815-2004). The ratio of concentrate to roughage of the diet was adjusted to 50:50. The roughage was oat hay. The concentrates mainly composed of corn, soybean meal, wheat bran, and premix. The feed ingredients and chemical composition of basal diet are presented in Table 1.

**Table 1:** Feed compositions and nutrient levels of the experimental diet (dry matter basis)

Ingredients, %		Nutrient levels, %	
Oat hay	50.00	NEg^b^, MJ/kg	3.49
Corn	38.90	CP	10.22
Wheat bran	1.50	EE	2.37
Soybean meal	2.00	NDF	41.69
Rape cake	2.50	ADF	23.11
Fermented distiller’s grains	2.50	Ca	0.57
Soybean oil	0.40	P	0.31
CaHCO_3_	0.05		
CaCO_3_	0.65		
NaHCO_3_	0.50		
Salt	0.50		
Premix^a^	0.50		

*NEg* net energy for gain, *CP* crude protein, *EE* ether extract, *NDF* neutral detergent fiber, *ADF* acid detergent fiber

^a^The premix provided following per kilogram of the diet: Co (as cobaltous chloride 6-hydrate) 0.12 mg, Cu (as copper sulfate) 11.67 mg, I (as calcium iodate) 0.58 mg, Fe (as ferrous sulfate) 58.33 mg, Mn (as manganese sulfate) 23.33 mg, Se (as sodium selenite) 0.23 mg, Zn (as zinc sulfate) 35 mg, VA 2 200 IU, VD 275 IU, VE 90 IU

^b^NEg was a calculated value; the other nutrient levels of the diet were measured values

### Sample collection

The BW of all cattle were measured on d 0 and 60 before morning feeding, and the average daily gain (ADG) was calculated from the initial and final BW. Accurate feed intake of each cattle was recorded daily and converted into dry matter intake (DMI). Feed efficiency was determined by dividing DMI by ADG. On d 60, blood samples were collected from all cattle via the jugular vein before morning feeding. Then, the serum samples were obtained by centrifugation at 3200*g* and 4 ℃ for 10 min. The serum samples were collected in 1.5 mL sterile microtubes and stored at − 20 °C for later analysis. On d 60, ruminal fluid samples were collected using a flexible esophageal tube (Anscitech Co., Ltd., Wuhan, Hubei, China) from all animals at 4 h after morning feeding. The first 150 mL of ruminal fluid samples were discarded in order to avoid the saliva contamination. Subsequently, the ruminal pH was measured immediately with a portable pH meter (Anscitech Co., Ltd., Wuhan, Hubei, China). The fluid samples were squeezed through 4 layers of cheesecloth and transferred into sterile tubes. A part of fluid samples were frozen immediately in liquid nitrogen and stored at − 80 °C for analyzing bacterial community. The other part of fluid samples were stored at − 20 °C for analysis of rumen fermentation.

Beginning at 00:00 on d 58, fecal samples (about 300 g) of all animals were collected at 6-h intervals for 3 d by stimulating the rectum to induce defecation. The sampling time on the following day was moved forward 2 h (12 samples in total). The specific sampling time points were as follows: (d 58: 00:00, 06:00, 12:00, and 18:00; d 59: 22:00, 04:00, 10:00, and 16:00 h; d 60: 20:00, 02:00, 08:00, and 14:00) (Ma et al. 2021). Meanwhile, the fresh basal ration and orts were collected daily. A part of fecal samples at every time point were frozen immediately in liquid nitrogen and stored at − 80 °C for analyzing bacterial community. Before analysis of bacterial community, the fecal samples from the same cattle were thawed and mixed, subsequently, the samples were used for high-throughput sequencing. Additionally, the daily diets, orts, and fecal samples were mixed, respectively by per cattle, subsampled, and stored at − 20 ℃ for analysis of nutrient digestibility.

### Samples analysis

Before analysis, the serum samples were thawed and thoroughly mixed. Subsequently, the concentrations of total protein (TP), albumin (ALB), urea nitrogen (UN), glucose (GLU), total cholesterol (TC), triglyceride (TG), and non-esterified fatty acid (NEFA) in serum were measured by a automatic biochemical analyzer (BS-280, Mindray Bio-Medical Electronics Co., Ltd., Shenzhen, Guangdong, China). Frozen ruminal fluid samples were thawed and centrifuged at 15,000*g* for 10 min at 4 °C, then the supernatant was analyzed for ruminal fermentation parameters, including volatile fatty acid (VFA) (Erwin et al. 1961) and ammonia N (Broderick et al. 1980).

The feces, diets, and orts were thawed and dried at 65 °C for 48 h to a constant weight. The dried samples were ground to pass through a 1-mm sieve (Anscitech Co., Ltd., Wuhan, Hubei, China). Subsequently, the contents of dry matter (DM), ether extract (EE), organic matter (OM), and crude protein (CP) in the feces, diets, and orts were analyzed according to the AOAC (2006) procedure. In addition, the neutral detergent fiber (NDF) and acid detergent fiber (ADF) contents were measured with the methods described by Van Soest et al. (Van Soest et al. 1991). The nutrient digestibility (D, %) was analyzed using the acid-insoluble ash (AIA) ratio technique. The contents of AIA in the feces (Af, %) and diets (Ad, %) were determined reference to Van Keulen and Young (1977). With the content of a nutrient in feces (Nf, %) and diet (Nd, %), the nutrient apparent digestibility was calculated with an equation as follows: D = [1 − (Ad × Nf)/(Af × Nd)] × 100.

### Ruminal and fecal bacterial community analytical procedure

Ruminal fluid and fecal samples of 5 cattle that were close to the group average BW in each group were detected by 16S rRNA gene sequencing. The main methods of ruminal and fecal bacterial DNA extraction and PCR amplification were followed the procedure described by our previous study (Ma et al. 2020c). Briefly, the ruminal fluid and fecal samples were used for total genomic DNA extraction via the TIANamp Stool DNA Kit (TIANGEN, Beijing, China). The 0.8% Agarose Gel Electrophoresis and a NanoDrop 2000 Spectrophotometer (Thermo Scientific, Waltham, MA, United States) were used to evaluate the concentration and purity of DNA. The universal primers 341F (5′-CCTACGGGRSGCAGCAG-3′) and 806R (5′-GGACTACHVGGGTWTCTAAT-3′) with 12 nt unique barcodes were used to amplify the V3–V4 variable region of the 16S rRNA gene from all DNA samples (Metzler-Zebeli et al. 2015). PCR products from all samples were pooled with equal molar amount for subsequent sequencing analysis. Sequencing libraries were generated using TruSeq DNA PCR-Free Sample Prep Kit (Illumina, San Diego, CA, USA) reference to the manufacturer’s instructions and index codes were added. The library were sequenced on an Illumina HiSeq 2500 platform (Illumina, San Diego, CA, USA) by 2 × 250 bp paired-end sequencing.

### Sequencing data analysis

The detailed methods of paired-end reads assembly, operational taxonomic units (OTUs) clustering, and taxonomy assignment were described in our previous study (Ma et al. 2020c). In order to avoid the effects of sequencing depth on community diversity, all samples were homogenized and the sample with the least amount of data was taken as the standard for re-sampling. The sequencing data analysis was conducted using R software (version 3.5.3) (R Core Team 2016). The Vegan was used to calculate the alpha- and beta- diversity parameters (Oksanen et al. 2016). Additionally, the rarefaction curves were generated based on the number of OTUs. The vegdist function of Vegan was used to calculate the distance of Bray–Curtis. The principal coordinates analysis (PCoA) was analyzed using the ape package based on Bray–Curtis dissimilarity matrices (Paradis et al. 2004), and the permutational multivariate analysis of variance (PerMANOVA) was calculated using the adonis function of Vegan. The heat map was drawn with the dominant bacteria using the z-score normalization for each sample [z score = (actual relative abundance of a genus − mean relative abundance of the same genus)/standard deviation].

### Statistical analysis

All data were performed normality and homogeneity of variances tests. Variables of growth performance, rumen fermentation, nutrient digestibility, serum parameters, alpha-diversity indexes, and bacterial relative abundance were analyzed by independent sample t-test of the SPSS statistical software (version 22.0 for Windows; SPSS, Chicago, USA), with each animal as an experimental unit. If the data did not satisfy normal distribution or homogeneity of variance, this data was analyzed using the non-parametric test of SPSS software. Data were shown as means and standard error of mean (SEM). A significance level was indicated at *P* < 0.05, and a trend was declared at 0.05 ≤ *P* < 0.10.

## Results

### Growth performance

The initial BW, final BW, DMI, and ADG of CAY group were higher (*P* < 0.05) than those of YAK group. Additionally, the DMI-to-ADG ratio in the CAY group was slightly lower (*P* = 0.062) as compared with the YAK group (Table 2).

**Table 2:** The differences of growth performance between yaks and cattle-yaks

Items	Groups		SEM	*P*-value
	YAK	CAY		
Initial BW, kg	179.30	208.00	7.013	0.011
Final BW, kg	210.00	247.80	8.819	0.004
DMI, kg/d	4.77	5.02	0.116	0.014
ADG, kg/d	0.51	0.65	0.034	0.005
FE	9.38	7.67	0.392	0.062

*BW* body weight, *DMI* dry matter intake, *ADG* average daily gain, *SEM* standard error of mean, *YAK* yaks (n = 10), *CAY* cattle-yaks (n = 10). FE = DMI/ADG

### Rumen fermentation

Ruminal pH was similar (*P* > 0.05) and averaged 6.59 and 6.61 in the YAK and CAY groups (Table 3). No significant difference (*P* > 0.05) of ammonia N concentration was observed between two groups. The concentrations of total VFA, acetate, and butyrate of CAY group were higher (*P* < 0.05) than those of YAK group. Furthermore, the CAY group exhibited a slightly higher (*P* = 0.062) propionate concentration as compared to YAK group. However, the acetate-to-propionate ratio displayed an opposite trend between two groups.

**Table 3:** The differences of rumen fermentation between yaks and cattle-yaks

Items	Groups		SEM	*P*-value
	YAK	CAY		
pH	6.59	6.61	0.081	0.910
Total VFA, mmol/L	69.92	80.47	2.350	0.013
Acetate, mmol/L	48.60	54.50	1.569	0.016
Propionate, mmol/L	13.20	16.22	0.553	0.062
Butyrate, mmol/L	8.12	9.75	0.351	0.009
Acetate-to-propionate ratio	3.71	3.36	0.016	0.088
Ammonia N, mg/dL	11.07	10.70	0.754	0.824

*VFA* volatile fatty acid, *SEM* standard error of mean, *YAK* yaks (n = 10), *CAY* cattle-yaks (n = 10)

### Nutrient digestibility

Notably, the apparent digestibility of DM and EE were not different (*P* > 0.05) between two groups (Table 4). However, the OM, NDF, and ADF digestibility of YAK group were higher (*P* < 0.05) than those of CAY group. Additionally, the CP digestibility in YAK group tended to be higher (*P* = 0.081) than that in CAY group.

**Table 4:** The differences of nutrient apparent digestibility between yaks and cattle-yaks (%)

Items	Groups		SEM	*P*-value
	YAK	CAY		
DM	77.32	73.93	1.347	0.227
OM	89.63	78.56	1.349	< 0.001
CP	71.95	64.25	2.224	0.081
EE	77.60	69.19	3.181	0.228
NDF	70.60	59.88	2.453	0.033
ADF	66.51	55.08	2.393	0.019

*DM* dry matter, *OM* organic matter, *CP* crude protein, *EE* ether extract, *NDF* neutral detergent fiber, *ADF* acid detergent fiber, *SEM* standard error of mean; *YAK* yaks (n = 10), *CAY* cattle-yaks (n = 10)

### Serum biochemical indexes

The contents of TP, ALB, UN, GLU, TC, TG, and NEFA were similar (*P* > 0.05) between YAK and CAY groups (Table 5).

**Table 5:** The differences of serum biochemical indexes between yaks and cattle-yaks

Items	Groups		SEM	*P*-value
	YAK	CAY		
TP, g/L	77.10	76.14	1.347	0.752
ALB, g/L	33.33	31.27	0.911	0.270
UN, mmol/L	5.28	5.70	0.302	0.573
GLU, mmol/L	5.09	4.95	0.138	0.677
TC, mmol/L	1.98	2.12	0.101	0.590
TG, mmol/L	0.34	0.34	0.024	0.940
NEFA, mmol/L	0.14	0.13	0.007	0.457

*TP* total protein, *ALB* albumin, *UN* urea nitrogen, *GLU* glucose, *TC* total cholesterol, *TG* triglyceride, *NEFA* non-esterified fatty acid, *SEM* standard error of mean, *YAK* yaks (n = 10), *CAY* cattle-yaks (n = 10)

### Microbial data acquisition and analysis

In the current study, 20 samples from the ruminal fluid and feces were collected from two groups. We obtained a total of 701,130 raw sequences by 16S rRNA gene sequencing, with an average of 35,057 ± 2927 (mean ± standard error) sequences per sample. After quality filtering of sequence, the effective sequences were obtained, with an average of 33,680 ± 2926 sequences per sample (Additional file 1: Table S1). The average sequencing length was 296 ± 2 bp. Based on a 97% nucleotide sequence identity between reads, the numbers of OTUs were 2589 and 2004 in the rumen and feces, respectively. The Q30 (Additional file 1: Table S1) and rarefaction curves (Additional file 1: Fig. S1) were generated for per sample to assess whether sampling provided sufficient OTUs coverage to accurately describe the bacterial community. In this study, the curves of all samples reached a plateau, indicating that a sufficient number of sequences were generated to explore bacterial diversity in the rumen and feces.

### Bacterial alpha- and beta-diversity analysis

Alpha-diversity analysis showed that the observed species, ACE, Chao1, Shannon and indexes in the rumen were similar (*P* > 0.05) between the YAK and CAY groups (Table 6). In the feces, no obvious difference (*P* > 0.05) of Chao1 and ACE indexes was observed between two groups. However, the observed species of CAY group was higher (*P* = 0.014) than that of YAK group. Additionally, the Shannon index of CAY group tended to be higher (*P* = 0.069) as compared to YAK group.

**Table 6:** Comparison of alpha-diversity indexes in the rumen and feces of yaks and cattle-yaks

Items	Groups		SEM	*P*-value
	YAK	CAY		
Rumen				
Observed species	1277.80	1355.80	41.892	0.430
Chao1	1540.92	1591.96	45.259	0.638
ACE	1538.92	1600.44	44.785	0.565
Shannon	6.02	6.17	0.090	0.497
Feces				
Observed species	897.80	996.60	22.190	0.014
Chao1	1175.18	1284.89	33.613	0.104
ACE	1220.33	1303.95	31.887	0.207
Shannon	5.38	5.74	0.101	0.069

*SEM* standard error of mean, *YAK* yaks (n = 5), *CAY* cattle-yaks (n = 5)

**Fig. 1:**
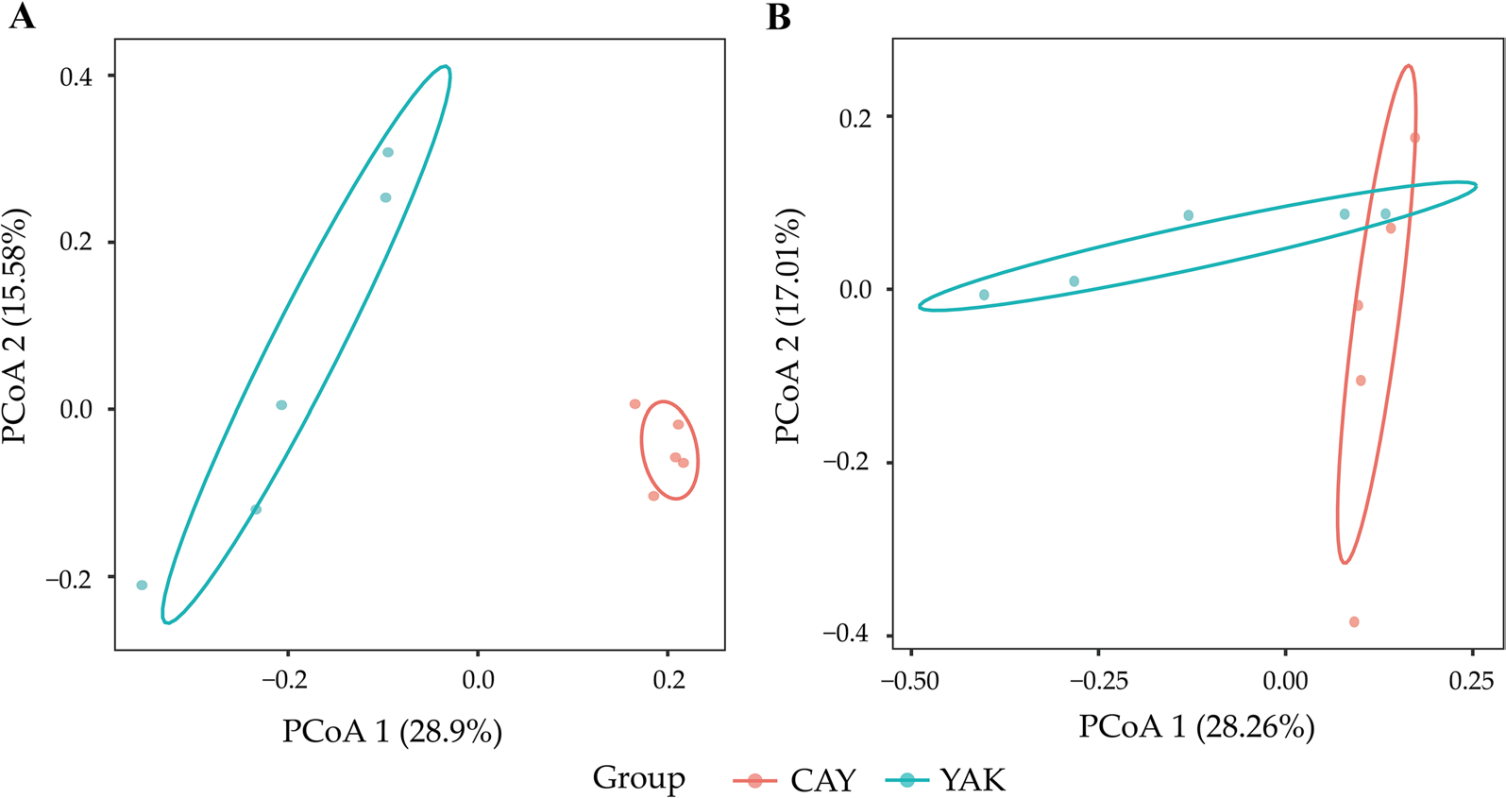
Principal coordinates analysis of bacterial communities in the rumen (**A**) and feces (**B**) between the YAK and CAY groups based on the Bray–Curtis distance. *YAK* yaks (n = 5), *CAY* cattle-yaks (n = 5)

In this study, based on Bray–Curtis dissimilarity matrices, when using PCoA to determine the structure of microbiota in the rumen and feces between the YAK and CAY groups, the bacterial communities in the rumen (Fig. 1A) and feces (Fig. 1B) were clearly separated from each other. Meanwhile, the PerMANOVA was used to evaluate whether there is a significant difference of distance between the YAK and CAY groups. The results revealed significant differences in the rumen (R^2^ = 0.273, *P* = 0.007) and feces (R^2^ = 0.204, *P* = 0.007) between two groups (Additional file 1: Table S2).

### Bacterial community in the rumen and feces

**Fig. 2:**
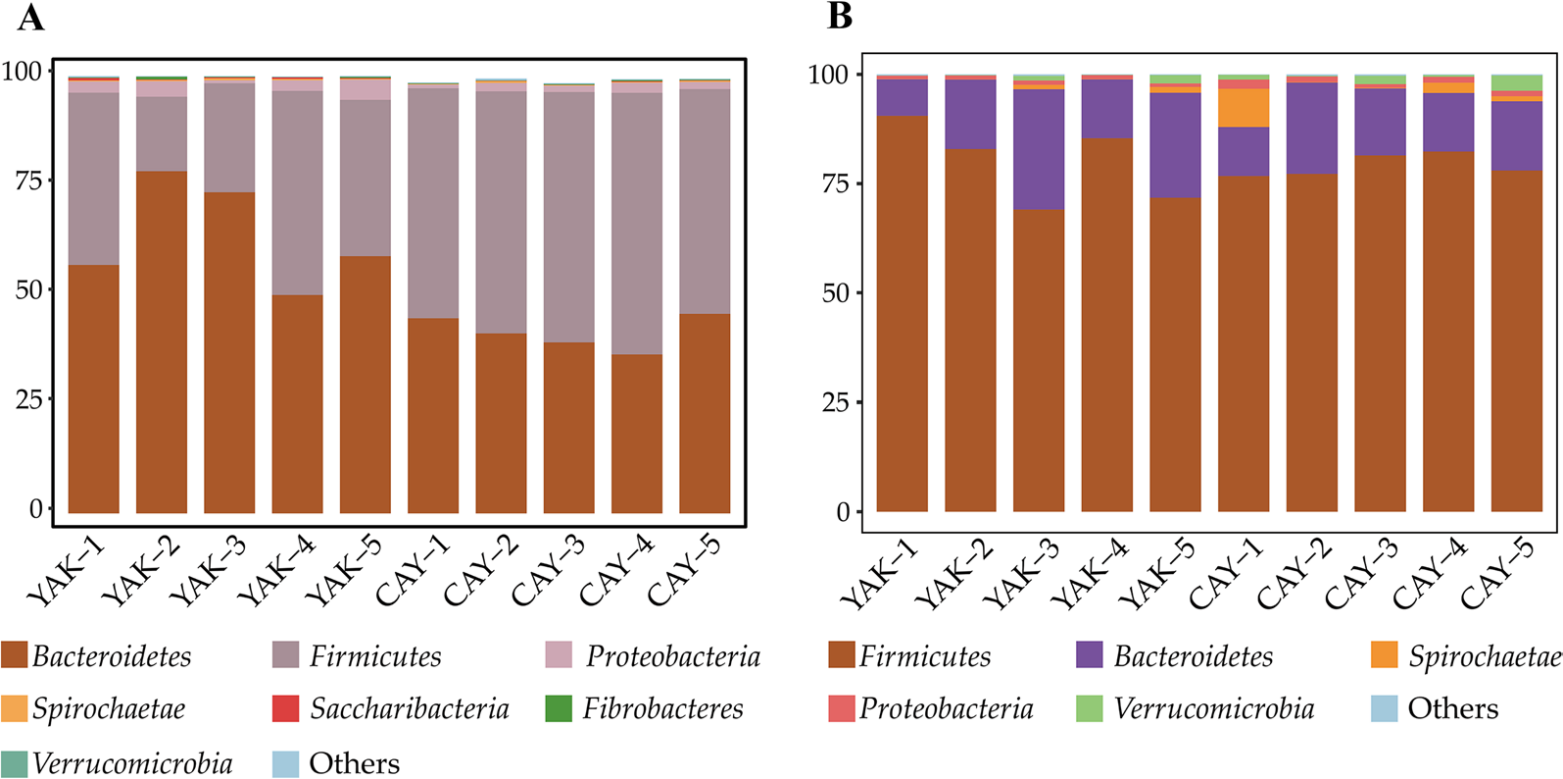
Bacterial composition at the phylum levels in the rumen (**A**) and feces (**B**) of yaks and cattle-yaks. *YAK* yaks (n = 5), *CAY* cattle-yaks (n = 5)

**Fig. 3:**
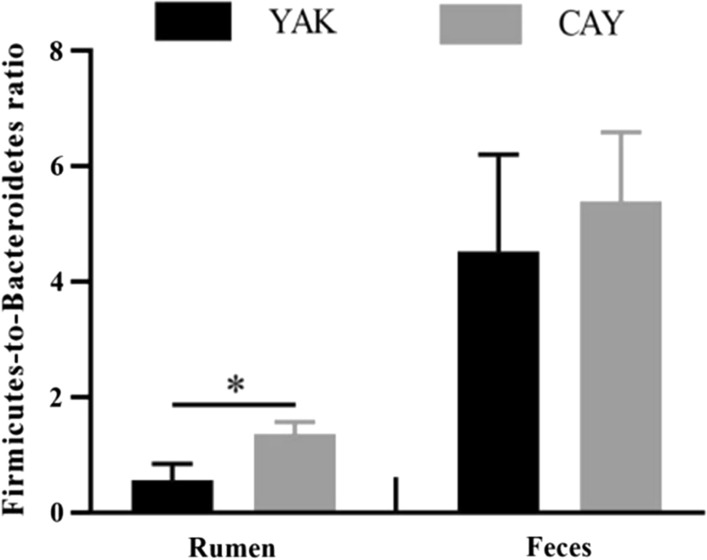
The difference of *Firmicutes*-to-*Bacteroidetes* ratio in the rumen (**A**) and feces (**B**) between the YAK and CAY groups. *YAK* yaks (n = 5), *CAY* cattle-yaks (n = 5). The asterisk indicates a significant difference between two groups (*P* < 0.05)

**Fig. 4:**
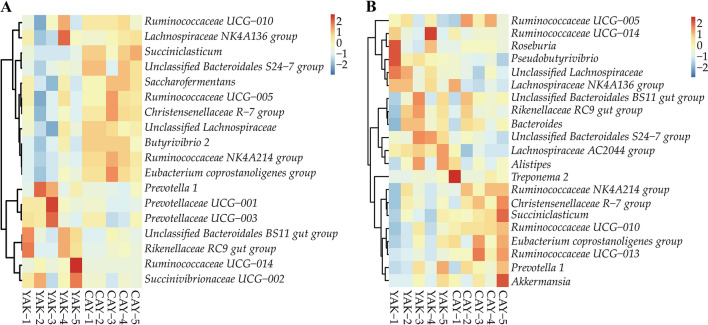
Heat map showing the relative abundance of dominant bacteria at the genus level in the rumen (**A**) and feces (**B**) between the YAK and CAY groups. *YAK* yaks (n = 5), *CAY* cattle-yaks (n = 5). The genus with the average relative abundance was ≥ 1% in at least one group

In the rumen, a total of 17 phyla were observed in 10 samples, of which *Bacteroidetes* (YAK = 66.37% and CAY = 41.28%), *Firmicutes* (YAK = 32.78% and CAY = 55.32%), and *Proteobacteria* (YAK = 2.75% and CAY = 1.62%) were the most abundant (Fig. 2A; Additional file 1: Table S3). Compared with YAK group, the relative abundances of *Firmicutes* and *Verrucomicrobia* were significantly increased (*P* < 0.05) in CAY group. However, the *Bacteroidetes* and *Saccharibacteria* showed an opposite trend between two groups. Furthermore, a higher (*P* < 0.05) *Firmicutes*-to-*Bacteroidetes* ratio (F/B ratio) was recorded in CAY group as compared to YAK group (Fig. 3). At the genus level, results showed that the predominant genera in the rumen of yaks and cattle-yaks mainly included *Prevotella 1* (YAK = 30.90% and CAY 16.48%), *Succiniclasticum* (YAK = 3.62% and CAY = 10.35%), *unclassified Bacteroidales BS11 gut group* (YAK = 8.39% and CAY = 5.34%), and *Rikenellaceae RC9 gut group* (YAK = 7.91% and CAY = 5.75%) (Fig. 4A; Additiona file 1: Table S3). The relative abundances of *Succiniclasticum*, *unclassified Bacteroidales S24-7 group*, *Ruminococcaceae NK4A214 group*, *Eubacterium coprostanoligenes group*, *Butyrivibrio 2*, and *Saccharofermentans* in CAY group were significantly higher (*P* < 0.05) than those in YAK group. Besides, the CAY group tended to have a higher relative abundances of *Christensenellaceae R-7 group* (*P* = 0.066) and *unclassified Lachnospiraceae* (*P* = 0.062) as compared with YAK group. However, the *Prevotella 1* and *Prevotellaceae UCG-001* relative abundances were higher (*P* < 0.05) in YAK group that those in CAY group.

In the feces, we identified 16 phyla from YAK and CAY groups. The majority of the bacteria belonged to the *Firmicutes* (YAK = 79.93% and CAY = 79.14%), *Bacteroidetes* (YAK = 17.85% and CAY = 15.33%), and *Spirochaetae* (YAK = 0.50% and CAY = 2.50%), accounting for more than 95% of all bacterial taxa (Fig. 2B; Additional file 1: Table S4). The relative abundance of *Proteobacteria* in CAY group was higher (*P* = 0.039) than that in YAK group. No obvious difference (*P* > 0.05) of other bacterial phyla was found between two groups. The F/B ratio was similar (*P* > 0.05) in the fecal sample between two groups (Fig. 3). At the genus level, the *Ruminococcaceae UCG-005* (YAK = 27.60% and CAY = 29.23%) was the most dominant bacterium in the feces of two groups, followed by *unclassified Lachnospiraceae* (YAK = 10.12% and CAY = 5.51%), *Rikenellaceae RC9 gut group* (YAK = 5.84% and CAY = 5.04%), and *Lachnospiraceae NK4A136 group* (YAK = 5.74% and CAY = 3.99%) (Fig. 4B; Additional file 1: Table S4). Compared with YAK group, the bacteria, including *Ruminococcaceae UCG-010*, *Ruminococcaceae UCG-013*, *Ruminococcaceae NK4A214 group*, and *Succiniclasticum*, were significantly enriched (*P* < 0.05) in CAY group. However, the *unclassified Lachnospiraceae*, *Lachnospiraceae NK4A136 group*, *Pseudobutyrivibrio*, *unclassified Bacteroidales S24-7 group*, and *Lachnospiraceae AC2044 group* were significantly enriched (*P* < 0.05) in YAK group. In addition, the relative abundance of *Eubacterium coprostanoligenes group* in CAY group was slightly higher (*P* = 0.089) than that in YAK group.

## Discussion

Under the condition of ensuring that different breeds of beef cattle are of similar age, it is difficult to meet the requirement of same weight. Our previous study found that in beef cattle, the BW of introduced breed was higher than that of local breed under the same age condition (Wang 2019). In the present study, the ADG and feed efficiency of CAY group were higher than those of YAK group. The reason may be that cattle-yaks has the heterosis created by the Simmental cattle genes. Cao et al. (2018) reported that the withers height, chest girth, and BW of 24-month-old cattle-yaks were higher than those of yaks with similar age, which was basically in line with our study. Blood biochemical indexes are associated with the health of animals. To a certain extent, the biochemical indexes can not only reflect the changes of organ function, but also reflect the metabolic status of body (Ma et al. 2021). The contents of TP, ALB, UN, and GLU in serum can be used to reflect the protein and energy metabolism, while TG and NEFA are key indexes of lipid metabolism (Ma et al. 2021). In the present study, no significant difference of serum parameters was observed between YAK and CAY groups, indicating that yaks and cattle-yaks were both in normal condition.

On the other hand, our study found that the YAK group showed higher apparent digestibility of NDF and ADF compared to CAY group. In general, the apparent digestibility of ruminants is influenced by the digestibility characteristics of diet and ruminal microbiota (Dschaak et al. 2011). In our study, all animals were fed the same diet. Previous studies have confirmed that the yaks possess a unique ruminal microbial ecosystem and the nitrogen utilization is more efficient than cattle living in low altitude areas (Long et al. 1999; Huang et al. 2012; Wang et al. 2016). In the rumen of yaks, when the feed resources are scarce, the microbes can efficiently degrade natural grass or withered grass to meet the nutritional requirement; besides, the microbes can produce higher activity of lignocellulose hydrolase, which are conducive to increasing the fiber digestibility (An et al. 2005). In the following study, we investigated the bacterial community in the rumen and feces of yaks and cattle-yaks.

The ruminal pH, which normally ranges from 6 to 7, can be used to evaluate the health condition of rumen. The ruminal pH affects the growth and proliferation of microbes, and then regulates the ruminal VFA production (Wales et al. 2004). In the current study, the ruminal pH of two groups was within the normal range of 6.59–6.61. Generally, the ruminal ammonia N concentration is 6 ~ 30 mg/dL (Preston et al. 1988). The concentration of ammonia N in YAK and CAY groups was within the normal scope. Shi et al. (2019) found that the ammonia N concentration in the rumen of yaks was higher than that of cattle-yaks in summer and winter. However, our study showed that the ruminal ammonia N concentration was similar between two groups. In Shi et al. (2019) study, the feed was different; but in our study, the diet was same. The VFAs are mainly produced by microbial degradation of carbohydrates in the rumen (He et al. 2018). As the main energy substrate, VFAs are rapidly absorbed by the ruminal epithelium and provide approximately 80% of energy requirements for ruminants (Gbel et al. 1997). In the current study, the concentrations of acetate, propionate, butyrate, and total VFA in CAY group were higher than those in YAK group. Therefore, the cattle-yak could obtain more energy than yak from the same diet, which was beneficial to promote the growth performance. Previous studies in vivo (Huang et al. 2012) and in vitro (Zhang et al. 2016) found that the total VFA concentration was higher in yaks than that in cattle under grazing condition. Another study reported that ruminants with higher DMI had higher level of VFA concentration in the rumen (Shi et al. 2019), which were consistent with our results. The lower acetate-to-propionate ratio provides faster and higher energy for the body, which is beneficial to improve the growth performance of animals (Ma et al. 2020a). The higher acetate-to-propionate ratio means better cellulose digestibility (Zhou et al. 2017). Our study found that the YAK group exhibited a slightly higher acetate-to-propionate ratio, which matched the fiber digestibility.

Gastrointestinal microbiota plays an important role in the metabolism of exogenous and endogenous substrates into nutrients which can be used directly by the host (Holmes et al. 2012). In the present study, we compared the bacterial community in the rumen and feces of YAK and CAY groups. Our study found that the phyla *Firmicutes* and *Bacteroidetes* were the dominant bacteria of rumen and feces in YAK and CAY groups. Consistent with our results, the 2 phyla were also identified to be presented abundantly in the gut of yaks (Xue et al. 2016; Cui et al. 2020), dairy cattle (Mao et al. 2015) and steers (Oliveira et al. 2013), suggesting the important functional role of *Bacteroidetes* and *Firmicutes* in the gut of ruminants. Furthermore, the PCoA results revealed that the bacterial composition and structure in the rumen were distinct between YAK and CAY groups. In accordance with our results, in beef cattle, Li et al. (2019b) found that the diversity and structure of ruminal bacteria were distinct among different breeds. Another study only showed differences in the structure of ruminal bacteria among different breeds, but the diversity index was similar (Li et al. 2019a). Therefore, host genotype might affect the ruminal microbiota to some extent. Unfortunately, at present, few studies were conducted to evaluate the effects of breed on the bacterial community in the feces of ruminants. Our study found that the diversity and structure of bacteria in the feces were different between yaks and cattle-yaks.

In ruminants, the ruminal bacteria digest the feedstuff and convert it into VFAs, microbial protein, and vitamins to meet the requirements for maintenance, growth, and health of the host and themselves (Zhu et al. 2020). Ma et al. (2020c) reported that *Firmicutes* had an important function in the process of energy absorption. Moreover, *Firmicutes* have been verified to be involved in the degradation of oligosaccharide as well as VFA production (Mao et al. 2015). Our previous study found that grazing yaks with low feed efficiency showed lower *Firmicutes* relative abundance in the rumen (Zou et al. 2019). The increased relative abundance of *Firmicutes*, which can result in higher F/B ratio, is related to higher feed utilization of cattle (Myer et al. 2015). In human study, the higher F/B ratio in the gut was confirmed to be associated with obesity (Turnbaugh et al. 2006). In our study, the CAY group exhibited higher relative abundance of *Firmicutes* and F/B ratio compared to YAK group, indicating that the cattle-yak had higher energy utilization, thereby providing more energy for host to improve the weight gain. The phyla *Saccharibacteria* is related to cellulose utilization, and regarded as the utilization bacteria in the plant structure polysaccharide cellulose (Opdahl et al. 2018). In addition, a study on sheep found that the relative abundance of *Saccharibacteria* was negatively correlated with ADG (Du et al. 2019). The current study found that compared to YAK group, the CAY group displayed lower relative abundance of *Saccharibacteria*. This may explain the phenomenon that the cattle-yaks have lower NDF digestibility and higher ADG.

At the genus level, in the current study, the relative abundances of *Prevotella 1* and *Prevotellaceae UCG-001* in YAK group was higher than those in CAY group. The bacteria belonging to *Prevotellaceae* play an essential role in the decomposition of protein, starch, and hemicellulose (Rubino et al. 2017). Results from our study revealed that the yaks had higher ability of CP and fiber degradation, which were consistent with the results of nutrient digestibility. In the rumen, the *Succiniclasticum* can provide energy for ruminants through converting succinate into propionate (Vangylswykm et al. 1995). The main butyrate-producing anaerobic bacteria are *Butyrivibrio* fibrisolvens strains, and the important characteristic of bacteria belonging to genus *Butyrivibrio* is the production of butyrate (Xiao et al. 2016). Moreover, the *Saccharofermentans* in the rumen can degrade the plant polysaccharide and the final fermentation products are acetate and propionate (Perea et al. 2017). In the present study, the CAY group showed higher relative abundances of *Succiniclasticum*, *Butyrivibrio 2*, and *Saccharofermentans* compared to YAK group, indicating that the cattle-yak could produce higher level of VFA, which were line with the results of rumen fermentation.

Previously, a research reported that *Bacteroidetes* and *Firmicutes* were the dominant bacteria in the feces of calves, accounting for more than 90% of the total bacteria (Meale et al. 2017). Consistent with previous research, our results showed that *Bacteroidetes* and *Firmicutes* were the dominant bacteria in the feces of YAK and CAY groups. *Proteobacteria* can be used as a microbial signature of dysbiosis in gut microbiota. When the relative abundance of *Proteobacteria* is higher, the gut may suffer from inflammation (Shin et al. 2015). In our study, the *Proteobacteria* relative abundance of CAY group was higher that that of YAK group. However, the relative abundance of *Proteobacteria* in the feces of yaks and cattle-yak was lower compared to the previous study in ruminants (Oliveira et al. 2013), which might be attributed to the living environment. Similar to the ruminal microbiota, in the hindgut of bovine species, the microbiota possess cellulase, deaminase, protease, and urease activities, and the fermentation products include VFA, ammonia N, and microbial cells. In dairy cattle, ~ 5–10% of total tract carbohydrate is degraded in the hindgut (Gressle et al. 2011; Stevens et al. 1980).

At the genus level, Mao et al. (2015) found that the dominant bacteria in the rectal contents of Holstein cows are *unclassified Ruminococcaceae*, *Peptostreptococcaceae*, and C*lostridium*, whereas Meale et al. (2017) reported that the *unclassified Ruminococcaceae* and *Bacteroides* were the main bacteria in the feces of weaned calves. The *Ruminococcaceae UCG-005* and *unclassified Lachnospiraceae* were the predominant bacteria in the current study, which might be due to the differences in diet and age of animals. The bacteria belonging to the *Lachnospiraceae* have been verified to generate the cellulase, which play an vital role in the decomposition of fiber in the gut (Li et al. 2019b). We found that the relative abundance of *unclassified Lachnospiraceae*, *Lachnospiraceae NK4A136 group*, and *Lachnospiraceae AC2044 group* in YAK group was higher, which was conducive to improving the fiber digestibility. The *unclassified Bacteroidales S24-7 group* can secrete carbohydrate degrading enzymes. In particular, it has been described as playing an important role in the degradation of polysaccharides, such as starch, hemicellulose and pectin (Ormerod et al. 2016). Compared with CAY group, the YAK group showed higher relative abundance of *unclassified Bacteroidales S24-7 group*, indicating that yak had higher ability of fiber degradation. In accordance with rumen, the CAY group showed higher relative abundance of *Succiniclasticum* compared to YAK group. A previous study reported that the beef cattle with high feed efficiency had higher relative abundance of *Ruminococcaceae* in the gut (Li et al. 2017). We found that the *Ruminococcaceae UCG-010*, *Ruminococcaceae UCG-013*, and *Ruminococcaceae NK4A214 group* relative abundances of CAY group were higher than those of YAK group. The results indicated that cattle-yak had higher feed efficiency, which matched the growth performance data. To sum up, the bacterial community showed obvious difference between yaks and cattle-yaks. However, the knowledge of the functional role of microbiota is still limited. In the future, meta-omics approaches should be used to further explore the microbial difference among different kinds of ruminants, especially animals living in plateau.

In the current study, the yaks showed higher digestibility of NDF and ADF, whereas the cattle-yaks displayed higher ADG and ruminal VFA concentrations. These results may be partly attributed to the difference of bacterial community in the gut. The *Prevotella 1*, *Prevotellaceae UCG-001*, *unclassified Lachnospiraceae*, *Lachnospiraceae NK4A136 group*, and *Lachnospiraceae AC2044 group* were significantly enriched in yaks, and the *Succiniclasticum*, *Butyrivibrio 2*, *Saccharofermentans*, and *Ruminococcaceae UCG-010* were significantly enriched in cattle-yaks.

## Supplementary Information

**Additional file 1**: **Table S1.** Data acquisition of all samples. **Table S2.** Analysis of PerMANOVA results of bacterial community according to ruminal fluid and fecal samples between YAK and CAY groups. **Table S3.** Comparison of the relative abundance (%) of the representative bacteria at the phylum and genus level in the rumen of yaks and cattle-yaks. **Table S4.** Comparison of the relative abundance (%) of the representative bacteria at the phylum and genus level in the feces of yaks and cattle-yaks. **Fig. S1.** Rarefaction curves for all ruminal and fecal samples.

## Data Availability

The sequences from the current study have been deposited in the NCBI Sequence Read Archive database with the accession number PRJNA714754.
