# Supplementary material for: Comparative study of growth performance, nutrient digestibility, and ruminal and fecal bacterial community between yaks and cattle-yaks raised by stall-feeding
Source: AMB Express. 2021 Jun 30;11:98. doi: 10.1186/s13568-021-01259-9 (PMC8245608; doi:10.1186/s13568-021-01259-9)
Supplement: Supplementary file 1 — Additional file 1: Table S1. Data acquisition of all samples. Table S2. Analysis of PerMANOVA results of bacterial community according to ruminal fluid and fecal samples between YAK and CAY groups. Table S3. Comparison of the relative abundance (%) of the representative bacteria at the phylum and genus level in the rumen of yaks and cattle-yaks. Table S4. Comparison of the relative abundance (%) of the representative bacteria at the phylum and genus level in the feces of yaks and cattle-yaks. Fig. S1. Rarefaction curves for all ruminal and fecal samples. [file 13568_2021_1259_MOESM1_ESM.docx]

**Table S1** Data acquisition of all samples

| **Items** | **Samples no.** | **Raw sequences** | **Effective sequences** | **Sequencing length** | **Q30%** |
| --- | --- | --- | --- | --- | --- |
| Rumen | YAK-1 | 33404 | 31876 | 298 | 93.40% |
|  | YAK-2 | 35308 | 31256 | 297 | 93.59% |
|  | YAK-3 | 34120 | 32341 | 298 | 93.37% |
|  | YAK-4 | 30250 | 29875 | 297 | 94.79% |
|  | YAK-5 | 39774 | 38670 | 297 | 93.63% |
|  | CAY-1 | 36560 | 35478 | 294 | 93.32% |
|  | CAY-2 | 32470 | 31234 | 294 | 94.32% |
|  | CAY-3 | 33816 | 32765 | 295 | 93.07% |
|  | CAY-4 | 31398 | 30267 | 295 | 94.20% |
|  | CAY-5 | 37148 | 36023 | 296 | 93.76% |
| Feces | YAK-1 | 37156 | 36134 | 294 | 93.47% |
|  | YAK-2 | 39610 | 38796 | 297 | 94.88% |
|  | YAK-3 | 35638 | 34347 | 294 | 94.09% |
|  | YAK-4 | 39878 | 38458 | 294 | 93.27% |
|  | YAK-5 | 31040 | 30123 | 294 | 94.09% |
|  | CAY-1 | 33968 | 32553 | 297 | 93.22% |
|  | CAY-2 | 35276 | 33950 | 297 | 93.58% |
|  | CAY-3 | 36232 | 33695 | 296 | 93.53% |
|  | CAY-4 | 36865 | 35569 | 298 | 94.13% |
|  | CAY-5 | 31219 | 30195 | 295 | 94.15% |

YAK, yaks (n = 5); CAY, cattle-yaks (n = 5); OTUs, operational taxonomic units.

**Table S2** Analysis of PerMANOVA results of bacterial community according to ruminal fluid and fecal samples between YAK and CAY groups

| **Regions** | **Groups** | **R^2^** | ***P*-value** |
| --- | --- | --- | --- |
| Rumen | YAK vs. CAY | 0.273 | 0.007 |
| Feces | YAK vs. CAY | 0.204 | 0.007 |

PerMANOVA, permutational multivariate analysis of variance; YAK, yaks (n = 5); CAY, cattle-yaks (n = 5). *P* < 0.05 means a significant difference of the bacterial structure in each corresponding regions between two groups.

**Table S3** Comparison of the relative abundance (%) of the representative bacteria at the phylum and genus level in the rumen of yaks and cattle-yaks

| **Level** | **Taxa** | **YAK** | **CAY** | **SEM** | ***P*-value** |
| --- | --- | --- | --- | --- | --- |
| Phylum | *Bacteroidetes* | 63.37 | 41.28 | 4.531 | 0.012 |
|  | *Firmicutes* | 32.78 | 55.32 | 4.562 | 0.011 |
|  | *Proteobacteria* | 2.75 | 1.62 | 0.381 | 0.148 |
|  | *Spirochaetae* | 0.30 | 0.24 | 0.038 | 0.431 |
|  | *Saccharibacteria* | 0.30 | 0.07 | 0.055 | 0.049 |
|  | *Fibrobacteres* | 0.20 | 0.11 | 0.054 | 0.417 |
|  | *Verrucomicrobia* | 0.09 | 0.15 | 0.016 | 0.043 |
| Genus | *Prevotella 1* | 30.90 | 16.48 | 2.363 | 0.028 |
|  | *Succiniclasticum* | 3.62 | 10.35 | 1.258 | 0.001 |
|  | *unclassified Bacteroidales BS11 gut group* | 8.39 | 5.34 | 1.266 | 0.268 |
|  | *Rikenellaceae RC9 gut group* | 7.91 | 5.75 | 0.734 | 0.181 |
|  | *unclassified Bacteroidales S24-7 group* | 3.38 | 5.62 | 0.522 | 0.020 |
|  | *Ruminococcaceae NK4A214 group* | 1.76 | 7.20 | 0.980 | 0.000 |
|  | *Ruminococcaceae UCG-005* | 3.49 | 4.86 | 0.452 | 0.139 |
|  | *Christensenellaceae R-7 group* | 2.89 | 4.91 | 0.559 | 0.066 |
|  | *Prevotellaceae UCG-001* | 4.55 | 1.71 | 0.501 | 0.043 |
|  | *Prevotellaceae UCG-003* | 2.05 | 1.65 | 0.231 | 0.423 |
|  | *Eubacterium coprostanoligenes group* | 0.94 | 2.45 | 0.295 | 0.002 |
|  | *Ruminococcaceae UCG-014* | 2.52 | 0.75 | 0.679 | 0.244 |
|  | *Butyrivibrio 2* | 0.98 | 2.20 | 0.253 | 0.004 |
|  | *unclassified Lachnospiraceae* | 1.28 | 1.706 | 0.117 | 0.062 |
|  | *Saccharofermentans* | 1.09 | 1.88 | 0.174 | 0.017 |
|  | *Succinivibrionaceae UCG-002* | 2.00 | 0.65 | 0.372 | 0.065 |
|  | *Ruminococcaceae UCG-010* | 1.03 | 1.26 | 0.116 | 0.342 |
|  | *Lachnospiraceae NK4A136 group* | 0.99 | 1.02 | 0.105 | 0.917 |

YAK, yaks (n = 5); CAY, cattle-yaks (n = 5); SEM, standard error of mean. The phylum with the average relative abundance was ≥ 0.1% in at least one group and The genus with the average relative abundance was ≥ 1% in at least one group.

**Table S4** Comparison of the relative abundance (%) of the representative bacteria at the phylum and genus level in the feces of yaks and cattle-yaks

| **Level** | **Taxa** | **YAK** | **CAY** | **SEM** | ***P*-value** |
| --- | --- | --- | --- | --- | --- |
| Phylum | *Firmicutes* | 79.93 | 79.14 | 2.008 | 0.861 |
|  | *Bacteroidetes* | 17.85 | 15.33 | 1.863 | 0.531 |
|  | *Spirochaetae* | 0.50 | 2.50 | 0.842 | 0.258 |
|  | *Proteobacteria* | 0.87 | 1.38 | 0.130 | 0.039 |
|  | *Verrucomicrobia* | 0.63 | 1.40 | 0.358 | 0.308 |
| Genus | *Ruminococcaceae UCG-005* | 27.60 | 29.23 | 0.929 | 0.413 |
|  | *unclassified Lachnospiraceae* | 10.12 | 5.51 | 1.172 | 0.040 |
|  | *Rikenellaceae RC9 gut group* | 5.84 | 5.04 | 0.400 | 0.347 |
|  | *Lachnospiraceae NK4A136 group* | 5.74 | 3.99 | 0.153 | 0.029 |
|  | *Christensenellaceae R-7 group* | 3.92 | 5.24 | 0.410 | 0.112 |
|  | *Pseudobutyrivibrio* | 3.81 | 1.72 | 0.468 | 0.014 |
|  | *Bacteroides* | 2.66 | 2.52 | 0.308 | 0.840 |
|  | *Ruminococcaceae UCG-010* | 1.91 | 3.27 | 0.289 | 0.007 |
|  | *Eubacterium coprostanoligenes group* | 1.64 | 3.03 | 0.410 | 0.089 |
|  | *Ruminococcaceae UCG-013* | 1.08 | 3.53 | 0.579 | 0.045 |
|  | *Ruminococcaceae NK4A214 group* | 1.73 | 2.17 | 0.114 | 0.041 |
|  | *unclassified Bacteroidales S24-7 group* | 2.29 | 1.35 | 0.211 | 0.015 |
|  | *Lachnospiraceae AC2044 group* | 2.50 | 0.99 | 0.296 | 0.002 |
|  | *Ruminococcaceae UCG-014* | 1.96 | 1.25 | 0.383 | 0.409 |
|  | *Treponema 2* | 0.50 | 2.50 | 0.843 | 0.259 |
|  | *Prevotella 1* | 1.16 | 1.35 | 0.176 | 0.612 |
|  | *Roseburia* | 1.28 | 0.87 | 0.221 | 0.415 |
|  | *Alistipes* | 1.17 | 0.88 | 0.187 | 0.465 |
|  | *Akkermansia* | 0.63 | 1.40 | 0.358 | 0.308 |
|  | *Succiniclasticum* | 0.77 | 1.25 | 0.116 | 0.025 |

YAK, yaks (n = 5); CAY, cattle-yaks (n = 5); SEM, standard error of mean. The phylum with the average relative abundance was ≥ 0.1% in at least one group and The genus with the average relative abundance was ≥ 1% in at least one group.


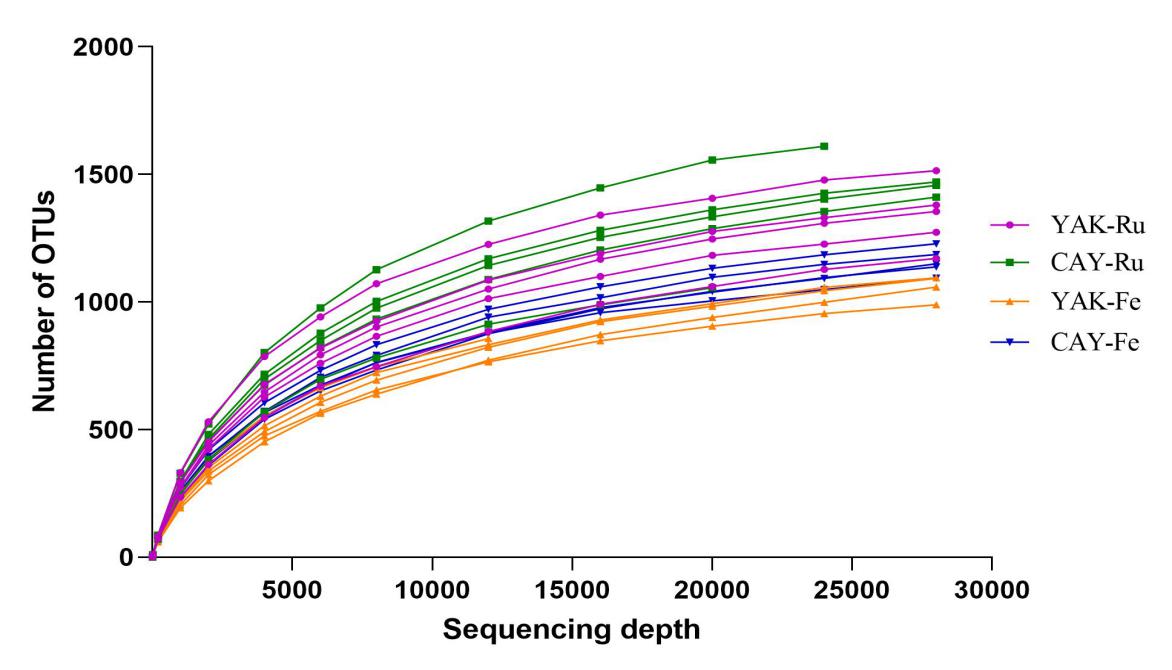


**Fig. S1.** Rarefaction curves for all ruminal and fecal samples (n = 20). Operational taxonomic units were assigned at the 97% sequence similarity level. YAK, yaks (n = 5); CAY, cattle-yaks (n = 5); Ru, rumen; Fe, feces.
